# The statistical impact of ROI referencing on quantitative susceptibility mapping

**DOI:** 10.1007/s10334-025-01226-6

**Published:** 2025-04-05

**Authors:** Patrick S. Fuchs, Oliver C. Kiersnowski, Carlos Milovic, Karin Shmueli

**Affiliations:** 1 Department of Medical Physics and Biomedical Engineering, University College London, London, UK; 2 Neuroradiology Unit, IRCCS Ospedale Policlinico San Martino, Genoa, Italy; 3 Biomedical Imaging Center, Department of Electrical Engineering, Pontificia Universidad Catolica de Chile, Santiago, Chile

**Keywords:** QSM, Susceptibility, Referencing, ROI, t test, ANOVA, Clinical application

## Abstract

In quantitative susceptibility mapping (QSM), it is impossible to define an absolute reference for the reconstructed susceptibility values. Therefore, it has been suggested to use a relative reference, such as the mean susceptibility within an anatomical ROI. We investigated the theoretical basis of referencing, and what impact it may have on statistical ROI comparisons, particularly for clinical applications. We analysed a clinical epilepsy study and in-silico QSM reconstruction challenge data with various reference regions. The results are analysed as in a clinical study and resulting statistical variations are investigated from a theoretical point of view. We found that referencing has an impact on the significance of clinical findings. These effects may arise from a change in the precision of test statistics due to referencing. We also show potential biasing of results from referencing. Our findings suggest there may not be one “optimal” reference region, and care should always be taken with reference region selection depending on the specific pathology or cohort under investigation. Not explicitly referencing is less likely to lead to false positives than cherry picking a reference region to maximize statistically significant results. We encourage results to be published with their reference to facilitate future comparisons of datasets from different sources.

## Introduction

The underlying tissue magnetic susceptibility ($\chi$) is related to the measured field perturbations from the gradient-echo phase ($\Delta B$) through a convolution with the magnetic dipole kernel (*D*) [1, 2].

1 $$\begin{aligned} \Delta B = \chi * D \end{aligned}$$

Inverting this forward model is known as quantitative susceptibility mapping (QSM), and, over the years, many different approaches have been published to achieve this [3–5]. The typical steps in the QSM pipeline involve echo combination, phase unwrapping, background field removal and dipole inversion.

In QSM, due to the dipole kernel having a zero at the origin in k-space (as part of a double conical surface of zeros), the scale of the DC offset, or zero frequency component is unknown. This is equivalent to having an unknown or undetermined mean susceptibility value across the whole image. Due to the background field removal step, QSM reconstructs a map of the bulk magnetic susceptibility of the tissue that is relative, not absolute. Therefore, the QSM reconstruction is equally valid for any scalar susceptibility offset [4].

It is not straightforward to invert the forward model due to the unit magnetic dipole kernel containing a double cone-shaped zero-valued surface, which leads to an undefined inverse. This ill-posed problem is typically solved through the application of regularisation, both implicit as well as explicit [5–14]. In 2019, a second QSM reconstruction challenge [15] was launched to compare susceptibility maps reconstructed using various QSM approaches to a ground truth (simulated) dataset, which enabled quantitative metrics to be applied. The metrics applied in the challenge are predominantly global image quality metrics, such as the normalised root mean squared error (nRMSE), or the susceptibility tuned similarity metric XSIM [16]. Pathological changes in magnetic susceptibility are, however, typically constrained to a local region e.g. specific anatomical structures in the brain, or regions of interest (ROI). Using the reconstruction challenge results, our recent preliminary work investigated the impact of the choice of reference ROI on different QSM reconstruction algorithms in selected regions of interest within the brain, such as: substantia nigra, red nucleus, thalamus [17].

Since clinical applications of QSM would typically use a t test or similar metric to investigate whether pathological changes are significant we also applied the t test on this ROI-based challenge analysis. We found that referencing strongly impacted the local accuracy of most reconstruction methods and could improve as well as degrade the susceptibility accuracy depending on the method and reference region used. We understand referencing to mean: subtracting the mean (or median) value within a clearly defined reference region from all values (i.e. the value in every voxel) in the reconstructed susceptibility map. The resulting map then represents susceptibility values *referenced to* the reference region’s mean or median susceptibility. The goal of referencing is usually to reduce inter-scan and inter-subject bias.

Although referencing of susceptibility maps has been suggested to obtain susceptibility values that are comparable between repeated measurements, subjects, and scanners [4], the relative nature of QSM makes choosing a reference challenging. Several studies have investigated potential reference regions [18–22]. There are two commonly accepted approaches to perform this referencing [4, 20, 23, 24]. The first is to set the mean of the whole reconstructed susceptibility map volume to 0 (equivalent to referencing to the mean susceptibility of the whole volume), which is often intrinsically done in the dipole inversion as the DC component of the reconstruction kernel is set to 0 and convolving with this kernel de-means the resulting image. The second involves computing the (mean) susceptibility in a region which is observed to have (almost) uniform susceptibility and referencing to the susceptibility in this region. Commonly used reference regions for QSM include the cerebrospinal fluid (CSF), or white matter regions such as the internal capsule. Cerebrospinal fluid is expected to have homogeneous isotropic susceptibility similar to water due to its (mostly) fluid nature. However, care must be taken to avoid including the choroid plexus when choosing a CSF ROI in the ventricles and to avoid partial volume effects (contamination with grey matter) if including CSF at the surface of the brain. For white matter regions, the apparent susceptibility depends on the fibre orientation with respect to the magnetic field, which can be a source of additional variability. The QSM consensus recommendations [4] suggest using two regions when there is uncertainty about an ideal reference region. If both choices result in agreement on the experiment, it can be assumed with more confidence that the result is not related to a referencing artifact.

Any inaccuracy in the QSM reconstruction of the reference region would lead to an error in the referenced ROI susceptibility values. We investigated whether such errors significantly impact the outcomes of clinical studies, and which reconstruction methods are susceptible to them.

In our preliminary ROI-based analysis of the QSM challenge phantom, we found that retrospectively referencing to the mean CSF susceptibility led to different results for different QSM reconstruction methods, improving the accuracy of some methods’ estimated susceptibility values while degrading others (due to bias or noise in reconstructing the reference region) against the ground truth (referenced in a similar fashion). Following this observation, our aims are to present a theoretical analysis of referencing, to investigate the influence of the dipole deconvolution method on the impact of referencing, and to visualise the influence referencing has on a clinical QSM study of temporal lobe epilepsy [25], evaluating the effects of referencing on this dataset. Finally, we present a novel referencing approach based not on an anatomical ROI but rather on a conceptual ROI defined according to the $R_2^{*}$ values of the tissue.

## Theory

Here, we analyse a clinical hypothesis, comparing mean susceptibility values of a specific ROI across groups of healthy and patient subjects. The samples are mean susceptibility values within an anatomical “test” ROI, and within a reference ROI (ref) in each subject in a group of n healthy controls and m patients, i.e.,

2 $$\begin{aligned} X_1,X_2,\ldots ,X_n\quad&\text {drawn from}\quad&N(\mu _\textrm{HC},\sigma _\textrm{HC} ^{2} )\nonumber \\ R_1^x,R_2^x,\ldots ,R_n^x\quad&\text {drawn from}\quad&N(\mu _\text {ref;HC},\sigma _\text {ref;HC} ^{2} )\nonumber \\ Y_1,Y_2,\ldots ,Y_m\quad&\text {drawn from}\quad&N(\mu _\textrm{patient},\sigma _\textrm{patient} ^{2} )\nonumber \\ R_1^y,R_2^y,\ldots ,R_m^y\quad&\text {drawn from}\quad&N(\mu _\text {ref;patient},\sigma _\text {ref;patient} ^{2} ) \end{aligned}$$

Here, $N(\text {mean},\text {variance})$ denotes a normal distribution within a group (across subjects), $\mu$ is the mean of the underlying distribution (“HC” for the healthy control group and “patient” for the patient cohort), and $\sigma ^{2}$ is the variance. If we want to test whether the susceptibility values in an ROI found in the patient cohort are statistically different from the healthy controls, our null hypothesis without referencing would be

3 $$\begin{aligned} H_0:\mu _\textrm{HC} =\mu _\textrm{patient}, \end{aligned}$$

whereas in the referenced case we could formulate our null hypothesis as:

4 $$\begin{aligned} H_0^\textrm{ref}:\mu _\textrm{HC} -\mu _\text {ref;HC} =\mu _\textrm{patient} -\mu _\text {ref;patient}. \end{aligned}$$

An important question arises from this formulation: Are these two hypotheses (and resulting statistical tests) equivalent? Two cases can readily be identified: one where the reference ROI susceptibility is uncorrelated with disease (across subjects), i.e. $\mu_{\textrm{ref};\textrm{HC} }=\mu_{\textrm{ref};\textrm{patient} }$. In this case, the two null hypotheses (and subsequent statistical tests) are equivalent. In the second case, when $\mu _{\textrm{ref};\textrm{HC} }\ne \mu _{\textrm{ref};\textrm{patient} }$, the hypotheses and resulting statistical tests are different. $\mu _\text {ref;HC} \ne \mu _\text {ref;patient}$ implies that there is a disease related change in the reference ROI’s magnetic susceptibility. This would bias the resulting susceptibility maps and change the null hypothesis and subsequent statistical tests. Even in the first case, $\mu _\textrm{ref;HC} =\mu _\textrm{ref;patient} \equiv \mu _\textrm{ref}$, when the null hypothesis is equivalent ($H_0^\textrm{ref} \equiv H_0$), test statistics can change due to referencing. Consider this illustrative example: a t-test statistic for two samples with unequal variance is

5 $$\begin{aligned} t_d=(\bar{x}_n-\bar{y}_m)/S_d, \end{aligned}$$

where $\bar{x}_n$ and $\bar{y}_m$ are the sample means of the healthy controls and patient cohort, respectively, and $S_d^{2}$ is the non-pooled variance i.e., the unbiased estimator for ${{\,\textrm{Var}\,}}(\bar{X}_n-\bar{Y}_m )$ for two samples with unequal variances:

6 $$\begin{aligned} S_d^{2}=(S_X^{2})/n+(S_Y^{2})/m. \end{aligned}$$

Here $S_X^{2}$ and $S_Y^{2}$ are unbiased estimators for $\sigma _X^{2}$ and $\sigma _Y^{2}$, respectively. The sample mean after referencing is the difference in sample means between the original values and the reference values, that is

7 $$\begin{aligned} \bar{x}_n^r=\bar{x}_n-\bar{r}_n. \end{aligned}$$

However, on referencing, the sample variance changes according to Bienaymé’s identity [26]

8 $$\begin{aligned} {{\,\textrm{Var}\,}}(X-R^x )={{\,\textrm{Var}\,}}(X)+{{\,\textrm{Var}\,}}(R^x )+2{{\,\textrm{Cov}\,}}(X,-R^x ). \end{aligned}$$

Note that ${{\,\textrm{Cov}\,}}(X,-R^x )= -{{\,\textrm{Cov}\,}}(X,R^x )$, (unlike ${{\,\textrm{Var}\,}}\left( -R\right) = {{\,\textrm{Var}\,}}\left( R\right)$, where the sign has no effect) thereby reducing the right-hand side on positive correlation between *X* and $R^x$. If the reference regions are uncorrelated with the susceptibility ROI of interest (${{\,\textrm{Cov}\,}}(X,-R^x )=0$) the variance of the referenced test is guaranteed to be larger than or equal to that of the original test. Similarly, this also holds for the variance of the referenced patient samples. In other words, referencing with an uncorrelated reference region increases the variance ($S_X^{2}$, $S_Y^{2}$ and, consequently, $S_d^{2}$) used in the t-test statistic. Note that a larger variance decreases the test statistic, thus decreasing the statistical power. It is important to note that the relevant covariance in Eq. (8) is between the mean susceptibility in the anatomical ROI and that in the reference ROI within each group. For example, the CSF is often used as a reference because it is a relatively large and uniform susceptibility region with low within-subject variance. However, the CSF across different subjects may display a larger variance ${{\,\textrm{Var}\,}}(R^x$). It is reasonable to imagine that there may be factors which lead to some subjects having a higher overall susceptibility throughout the brain than other subjects (such as age-related iron accumulation, unrelated de-myelination or a strong calcification). (In fact, referencing is designed to remove such inter-subject susceptibility variations to ensure comparability). In this case (of small inter-subject susceptibility variations), we may expect a small positive correlation between the mean susceptibility values in the test and reference ROIs (i.e. a small positive ${{\,\textrm{Cov}\,}}(X,R^x )$). To understand the effect on the t-test statistic of any covariance or correlation between the mean susceptibilities in the anatomical or “test” ROI and the reference ROI in each group, we start from the fact that the covariance is bounded by

9 $$\begin{aligned} -\sqrt{{{\,\textrm{Var}\,}}(X){{\,\textrm{Var}\,}}(R^x ) }\le {{\,\textrm{Cov}\,}}(X,R)\le \sqrt{{{\,\textrm{Var}\,}}(X){{\,\textrm{Var}\,}}(R^x ) }. \end{aligned}$$

If we assume equal variance of the test ROI and reference ROI ($\sigma _\textrm{HC} ^{2}=\sigma _\text {ref;HC} ^{2}=\sigma ^{2}$), this simplifies to

10 $$\begin{aligned} -\sigma ^{2}\le {{\,\textrm{Cov}\,}}(X,R^x )\le \sigma ^{2}, \end{aligned}$$

which leads to a referenced variance of

11 $$\begin{aligned} {{\,\textrm{Var}\,}}(X-R^x )&=\sigma _\textrm{HC} ^{2}+\sigma _\text {ref;HC} ^{2}-2{{\,\textrm{Cov}\,}}(X,R^x )\nonumber \\ {{\,\textrm{Var}\,}}(X-R^x )&=2\sigma ^{2}-2{{\,\textrm{Cov}\,}}(X,R^x ). \end{aligned}$$

This bounds the referenced variance to

12 $$\begin{aligned} 0\le {{\,\textrm{Var}\,}}(X-R^x )\le 4\sigma ^{2}, \end{aligned}$$

To be precise, the referenced variance could increase fourfold or decrease to 0 (in which case the mean susceptibility in the reference region can be shown to be equal to that in the test ROI). Some correlation is expected as, if the reference is to be used as a baseline, that baseline should shift the susceptibility means between subjects in turn as well. This means that the final variance is expected to be less than the sum of the separate variances ($2\sigma ^{2}$ in the case of equal variances, as above). In our experiments we have found the variance after referencing to be slightly higher than the original variance (${{\,\textrm{Var}\,}}(X-R^{x} )>\sigma ^{2}$).

## Methods

We investigated referencing in a synthetic dataset (from the 2019 QSM reconstruction challenge [15]) that has a ground truth, as well as on a cohort of patients with temporal lobe epilepsy and healthy controls originally investigated by Kiersnowski et al. [25]. The QSM challenge dataset has a single realistic numerical phantom reconstructed with many different QSM techniques which limited comparisons to those between reconstructions. Using this dataset, the aim was to highlight the influence of reconstruction method on the impact of referencing. On the other hand, the epilepsy cohort contains multiple subjects with both left and right temporal lobe epilepsy (LTLE and RTLE, respectively), as well as healthy controls (HC), allowing us to evaluate the effect of referencing in a published clinical QSM study.

### Reference regions

We considered four reference regions commonly used in the QSM literature [4, 19, 20, 24] and one novel referencing approach:

1. CSF: Cerebrospinal fluid,
2. WM: Corpus callosum (CC). For the epilepsy dataset, both the corpus callosum (CC) and internal capsule (IC) were chosen as white matter reference regions [18].
3. Whole brain: Whole brain (masked)

These three are common reference regions based on brain anatomy [18, 20]. These were obtained by brain segmentation, described in detail below for the specific datasets.

The final two reference regions are based on maps of the relative variance of the susceptibility maps across subjects and on subject-specific maps of the $R_2^{*}$ relaxation rate:

4. RelVar: Thresholding the relative susceptibility variance map [19] has been used as a method to provide a reference region independent of anatomical segmentation.

The relative susceptibility variance map was computed according to

13 $$\begin{aligned} \text {Relative Variance}\left[ \textrm{r}\right] =\frac{{{\,\textrm{Var}\,}}(\text {susceptibility} )[r]}{\textrm{mean}(\text {susceptibility} )[r]} \end{aligned}$$

where [r] indicates that these are values in a voxel at position r within a study-specific susceptibility template. This study-specific QSM template was generated, according to Acosta-Cabronero et al. [19], by registering the susceptibility maps in each subject to a study-specific $T_1$-weighted atlas, created by co-registering $T_1$-weighted images of all subjects together. The rationale for this method is that voxels with a low susceptibility variance across subjects are likely to provide a robust reference susceptibility. We chose the voxels with the lowest third percentile of relative variance because that resulted in a reasonably contiguous region of white matter as described in [19]. The reference susceptibility value was then calculated as the mean susceptibility in all voxels below the chosen threshold.

5. $R_2^{*}$-based: Thresholding the subject-specific $R_2^{*}$ map is our novel approach to obtain a reference region independent of anatomical segmentation.

Voxels with low $R_2^{*}$ values are expected to also contain few (or weak) susceptibility sources, and mostly liquid (isotropic) tissue (such as the CSF). Using a low relaxation rate as a cut-off then limits the potential contamination of grey matter voxels or partial volume effect which would be possible when using the CSF in the ventricles based on anatomical markers. We used auto-regression on linear operations (ARLO [27]) to compute the $R_2^{*}$ maps, with the reference “region” defined as those voxels with $R_2^{*}$ values below 4 Hz. We chose this threshold as low as possible while keeping a similar number of voxels to the more conventional, anatomical, reference regions.

### QSM reconstruction challenge 2.0 dataset

This is a synthetic dataset, which was used to compare and evaluate QSM reconstruction methods and is now used frequently as a ground-truth dataset for developing new reconstruction methods. More than 100 different reconstructions were submitted and compared in the original publication [15]. In this dataset, we used pairwise t tests to compare the reconstructed ROI mean susceptibility values with and without referencing to the ground truth susceptibility values to decide whether the values were reconstructed accurately. We compare overall accuracy for all submissions [28], and provide literature references to 11 frequently used and top scoring algorithms in Table 1 for convenience.

**Table 1:** Overview of major reconstruction methods compared in this ROI based analysis. DOIs are provided for convenience and correspond as closely as possible to the submissions, but there may be minor differences in implementation of the method for the challenge compared to the original publication. For further details we would like to refer to the original challenge results

**Method**	**Reference**
L1-QSM	DOI: 10.1002/mrm.28957
FANSI	DOI: 10.1002/mrm.27073
TFI	DOI: 10.1002/mrm.26331
MEDI	DOI: 10.1002/mrm.24272
FINE	DOI: 10.1016/j.neuroimage.2020.116579
SFCRe	DOI: 10.1109/TMI.2016.2544958
DeepQSM	DOI: 10.1016/j.neuroimage.2019.03.060
QSMNet	DOI: 10.1016/j.neuroimage.2020.116619
Star-QSM	DOI: 10.1002/nbm.3383
dirTik	DOI: 10.1002/jmri.24365
TKD	DOI: 10.1002/mrm.22135, 10.1002/mrm.24405

dirTik: direct Tikhonov regularized inversion

FANSI: FAst Non-linear Susceptibility Inversion

FINE: fidelity-imposed network edit

L1: L1-norm error sparsity promoting data fidelity term

SFCR: structural feature based collaborative reconstruction

TFI: total field inversion

TKD: thresholded k-space division

QSM: quantitative susceptibility mapping

The QSM challenge 2.0 ground truth ROIs are not separated per hemisphere and some commonly investigated deep gray matter regions are not divided into sub-regions e.g., pallidum, amygdala and hippocampus. Therefore, for this study, we re-segmented the phantom using the ground truth susceptibility maps combined with the anatomical contrast using MRCloud [29]. The relative variance was computed across all reconstructions, selecting the voxels within the brain that the reconstructions mostly “agreed” on. And for the $R_2^{*}$ based ROI the magnitude data provided with the challenge was fitted in the same way as the epilepsy data.

Anatomical test ROIs were chosen to be the same as those used in the epilepsy study: cerebrospinal fluid (CSF), corpus callosum (CC), internal capsule (IC), amygdala, caudate, pallidum, putamen, thalamus, and hippocampus.

Any inaccuracy in the QSM reconstruction of the reference region would lead to an error in the referenced ROI susceptibility values. We investigated whether such errors significantly impact the outcomes of clinical studies, and which reconstruction methods are susceptible to them.

A drawback of the synthetic QSM challenge dataset being a single “subject” is that all the reconstructions are based on the same data and, therefore, cannot be treated as independent measurements, making any group-wise statistics flawed. However, it is possible to compare the reconstructions to the ground truth, performing the same t test as described above, but considering the voxels within an ROI to be independent samples from an ROI-specific distribution (rather than ROI mean values being samples of a distribution across subjects).

**Fig. 1:**
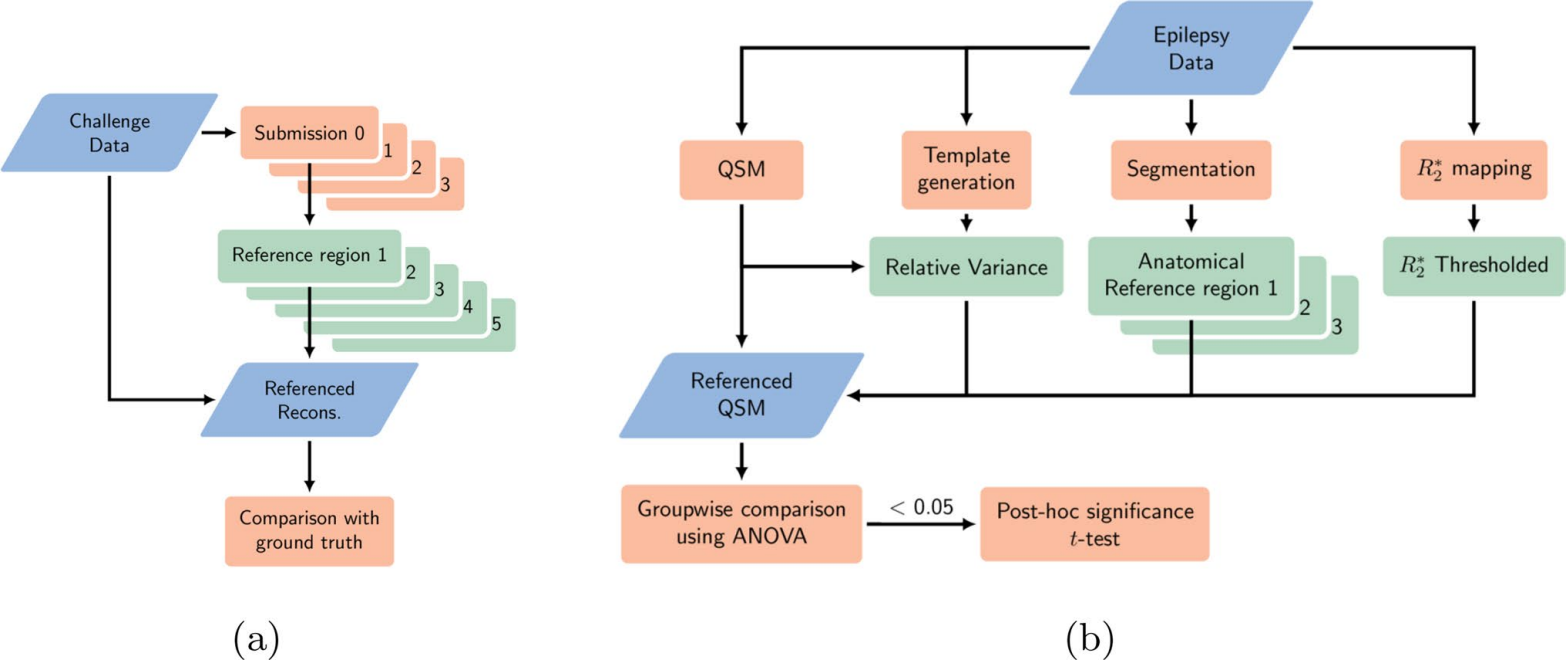
Illustration of Study design for experiments on **a** QSM challenge 2.0 simulated data and **b** Data from the epilepsy study [25] (right)

Note that in these comparisons we subtracted a scalar reference value from all the test ROI mean susceptibility values, which moved the test ROI means closer or further apart but did not impact the overall distribution of the voxel values within the test ROI. A visual depiction of this can be seen in Fig. 1a.

### Epilepsy dataset

To investigate the effect of referencing in the presence of pathology, we used a dataset of left and right temporal lobe epilepsy patients (LTLE and RTLE, respectively). The dataset, originally analysed by Kiersnowski et al. [25], consists of 27 healthy controls (HC), 19 patients with LTLE, and 17 with RTLE, with ages ranging from 16 to 67 years old (Range (Median); HC: 16.5$-$55.1 (30) LTLE: 19.4$-$66.5 (32.9) RTLE: 21.4$-$67.1 (34)).

The original results were not explicitly referenced and, hence, intrinsically used referencing method 3 from above (whole brain ). To validate this, we have included both unreferenced as well as explicitly referenced results. Therefore, to investigate the effect of referencing, here we compare the unreferenced results with results explicitly referenced to the regions 1-5 defined above. In each case, the referencing was performed on the “raw” susceptibility maps before age correction was applied. Following referencing and age-correction, we assessed groupwise ROI mean susceptibility differences using an analysis of variance ANOVA [30]. A post-hoc Tukey-Kramer multiple comparison of the subject ROI mean susceptibilities was then used to compute the statistical significance of the between-group differences. To segment test ROIs and reference regions 1-3, GIF [31–33] was used to segment the $T_1$ weighted images, HippoSeg [34] was used for the hippocampus, and the cerebrospinal fluid (reference region 1) was segmented using SPM12 [35]. Anatomical test ROIs included the amygdala, caudate, putamen, globus pallidus (internal and external combined), thalamus, internal capsule and hippocampus, for both hemispheres as the pathology is primarily single-sided, and these ROIs were also investigated in the original study (Fig. 3). As in the original analysis [25], all segmented ROIs were eroded by applying a spherical kernel of radius 1 (voxel) three times to the binary ROI mask to reduce partial volume effects, after which outliers (values outside the 1st and 99th percentile) were removed before computing the ROI statistics (mean and standard deviation). A visual depiction of the study design can be seen in Fig. 1b.

## Results

### Reference regions

**Fig. 2:**
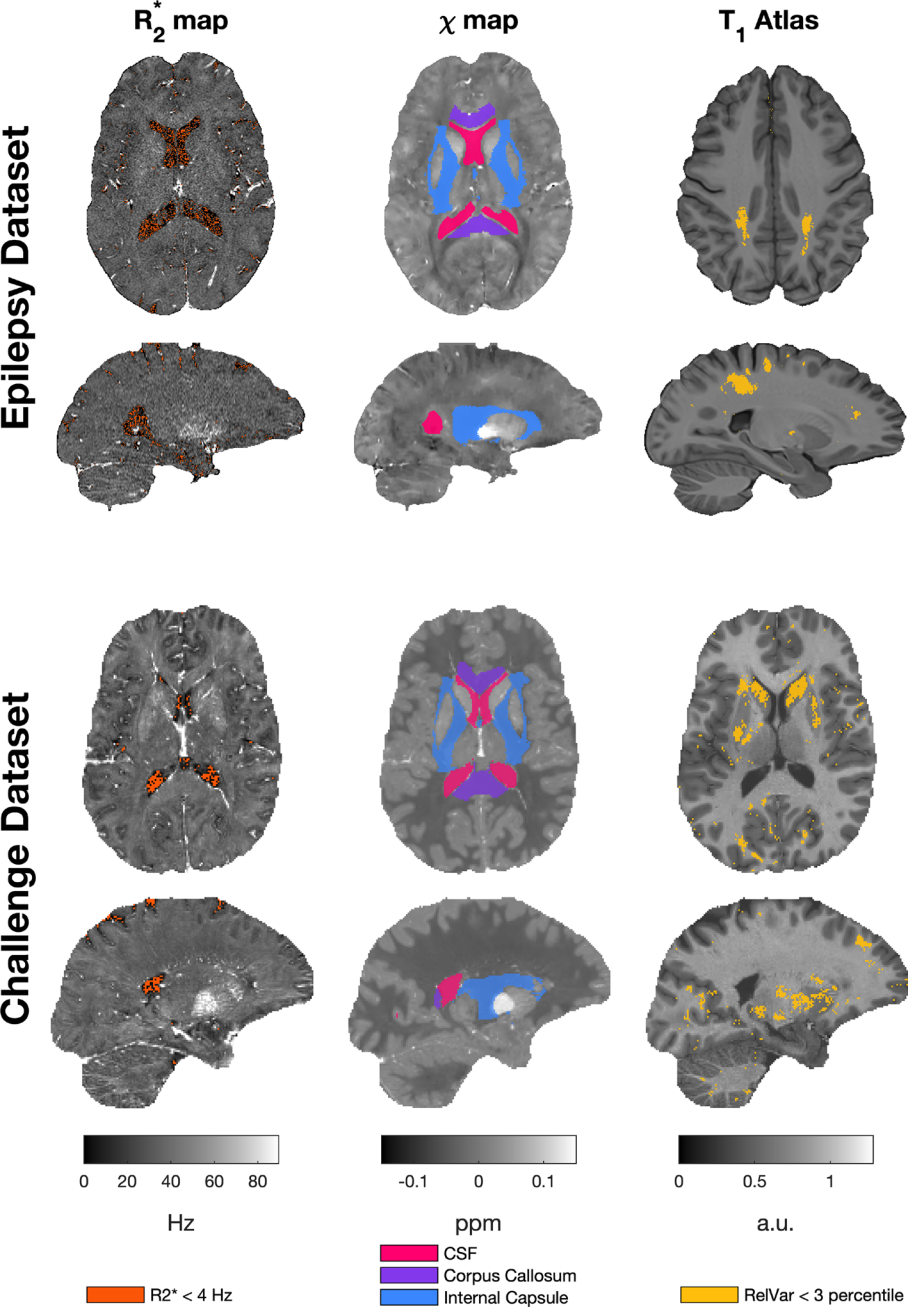
Reference regions overlayed on the Epilepsy dataset (representative subject, top) and QSM challenge 2.0 (Sim2SNR1) data (bottom). $R_2^{*}$ map (left) with corresponding susceptibility ground truth (middle), and $T_1$ weighted image (right). The segmentation was performed on the $T_1$ weighted image using GIF, and the relative variance determined across all the submitted reconstructions in the challenge dataset

**Fig. 3:**
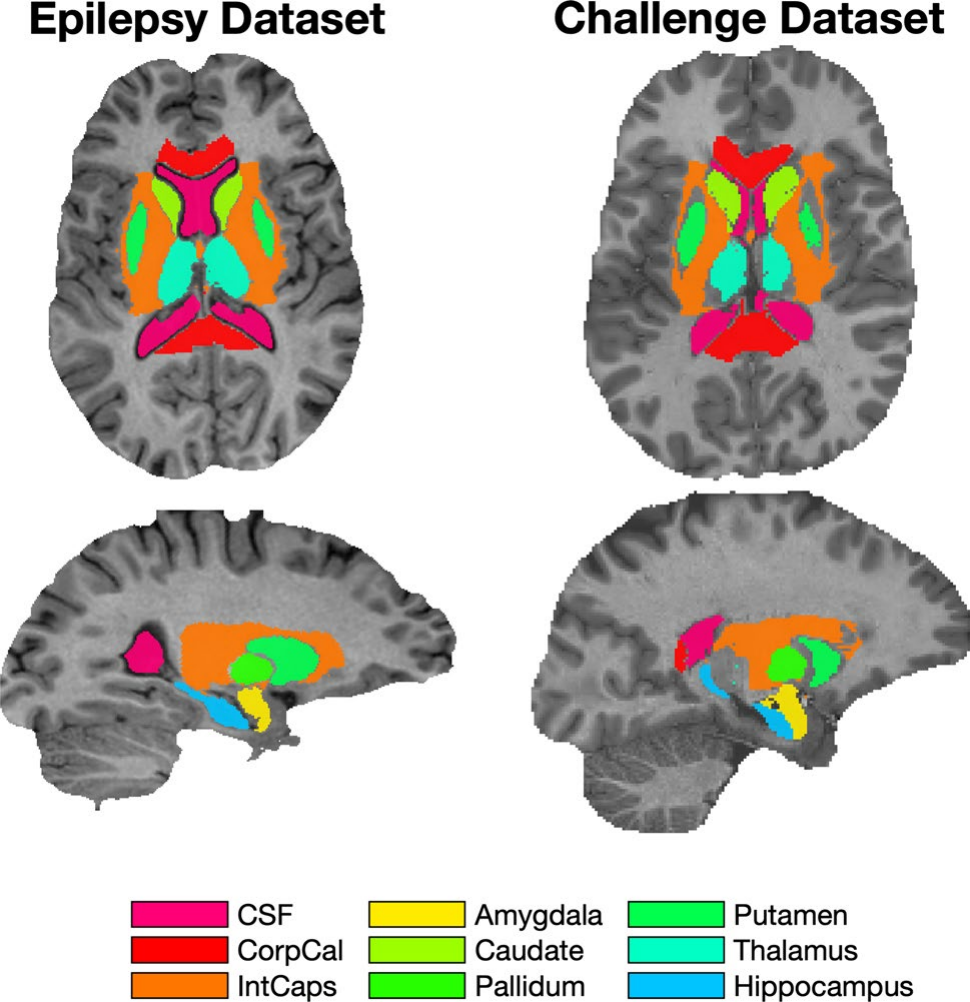
Deep gray matter test regions typically used in clinical magnetic susceptibility studies. Overlayed on $T_1$ weighted images of both datasets. The segmentations were performed using GIF and Hipposeg as described in the text

The anatomical test and reference ROIs are illustrated in the QSM challenge synthetic dataset and in a representative subject of the epilepsy study in Figs. 2 and 3. Voxels with a low relative variance (<3^rd^ percentile) are found in the posterior white matter, for example.

In Fig. 2 a representative $R_2^{*}$ based reference region (thresholded at 4 Hz) can be seen overlaid on an $R_2^{*}$ map. It largely consists of voxels containing cerebrospinal fluid, but it encompasses a larger region of the brain, often also selecting small pockets of CSF that would not usually be part of an anatomically defined reference ROI (constrained to the ventricles) [22]. Additionally, this map does not contain the CSF as a contiguous region but rather selects “specks” of CSF with low $R_2^{*}$ values (Fig. 2). Figure 3 shows the test regions of interest for both datasets, used in the subsequent analysis.

### QSM reconstruction challenge 2.0 dataset

**Fig. 4:**
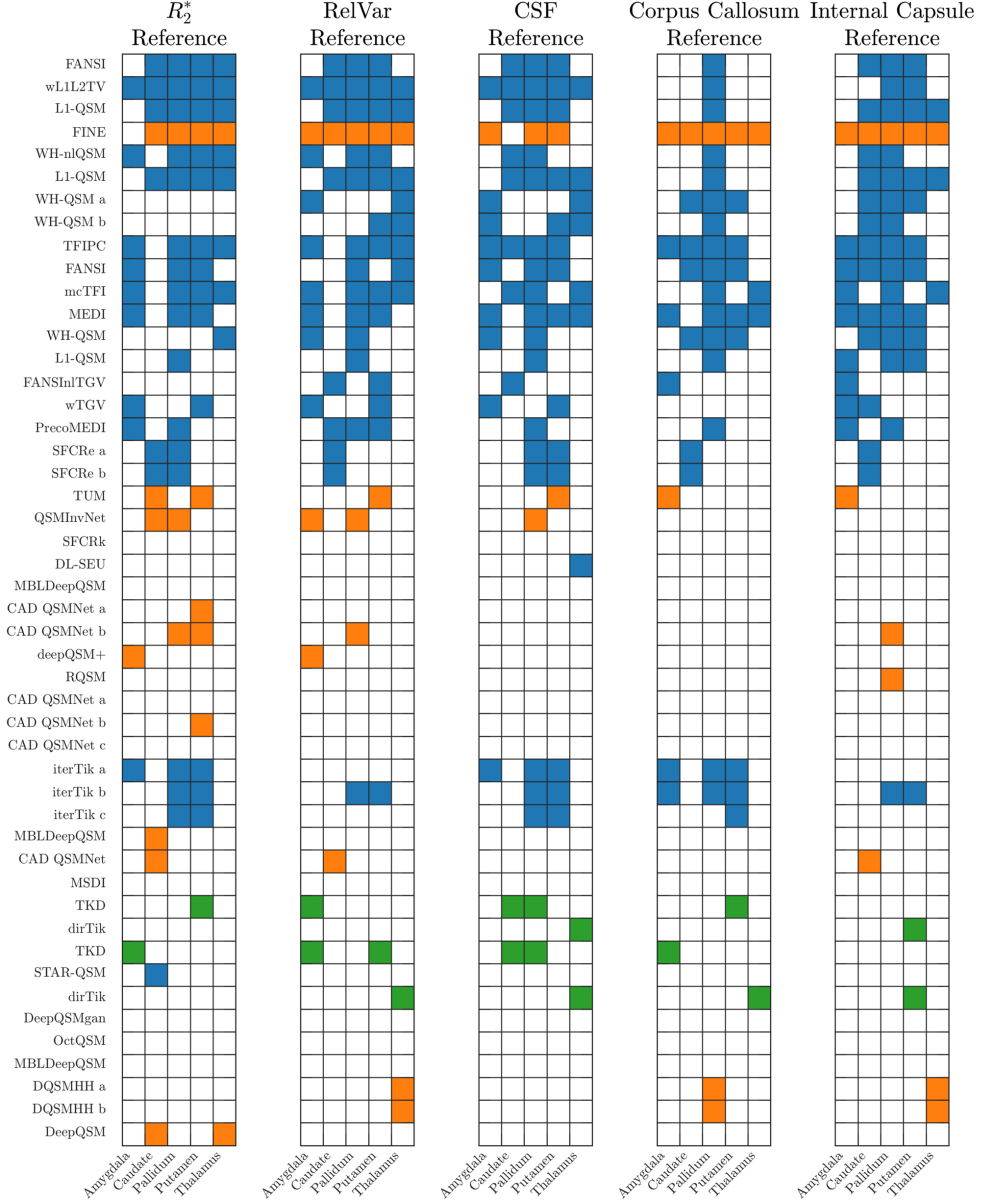
Accurately reconstructed regions of interest for the various reference methods for all tested reconstruction methods. Filled in squares are not significantly different from the ground truth. The colours refer to reconstruction type, blue for iterative methods, orange for machine learning-based methods, and green for direct reconstructions. Some methods appear multiple times, this is due to multiple submissions sharing the same “preferred acronym”. Since the reconstructions are ordered by global normalised mean square error (as can be found in the results table made publicly available by the challenge organisers), this can be used to look up what the specific differences between the submitted reconstructions (if any) is

T-test results for the challenge dataset are shown in Fig. 4. The colour in these plots is used to reflect the type of reconstruction algorithm, blue for iterative optimization methods, orange for deep learning methods and green for direct dipole inversion methods. Accurately reconstructed ROIs are defined as those whose (referenced) ROI mean susceptibility value is not statistically significantly different from its ground truth susceptibility value with 95% confidence. A summary of the number of accurately reconstructed ROIs based on reference region is presented in Table 2.

Clearly, reconstruction methods that perform better globally (i.e. are at the top of the list ordered by global normalised mean squared error) also accurately reconstruct the means of the test ROIs. Referencing to the corpus callosum is detrimental for many of the top-scoring algorithms (except FINE) but does not influence all reconstruction methods equally. This suggests that some reconstruction methods are more sensitive to referencing than others.

From Table 2, the anatomical reference regions outperform the other reference regions. However, the reference ROIs are also included as test ROIs in this sum, and referencing to the mean of these enforces an accurate estimate for all 48 submitted reconstructions, adding 48 accurate ROIs to the total sum.

**Table 2:** Table of the number of accurately reconstructed ROIs in the QSM challenge dataset for each reference region. Note that the CSF and WM regions are accurate in all 48 reconstructions, as expected when these regions are used as reference regions

ROI	None	${R_2^{*}}$	Relative Variance	Whole Brain	CSF	CC	IC
CSF	2	4	2	3	$48^{\dagger }$	6	1
Corpus Callosum	1	1	0	2	6	$48^{\dagger }$	4
Internal Capsule	6	4	11	3	4	6	$48^{\dagger }$
Amygdala	2	6	6	7	7	4	4
Caudate	2	7	5	4	2	2	4
Pallidum	7	8	11	10	12	11	16
Putamen	6	11	8	7	5	4	4
Thalamus	1	3	4	3	3	2	1
Hippocampus	0	0	0	0	0	0	0
Sum	27	44	47	39	87	83	82

ROI: region of interest; CSF: cerebrospinal fluid (ventricles); CC: corpus callosum;

IC: internal capsule

$^{\dagger }$ Reference ROI is accurate for all submissions upon referencing to it

### Epilepsy dataset

**Fig. 5:**
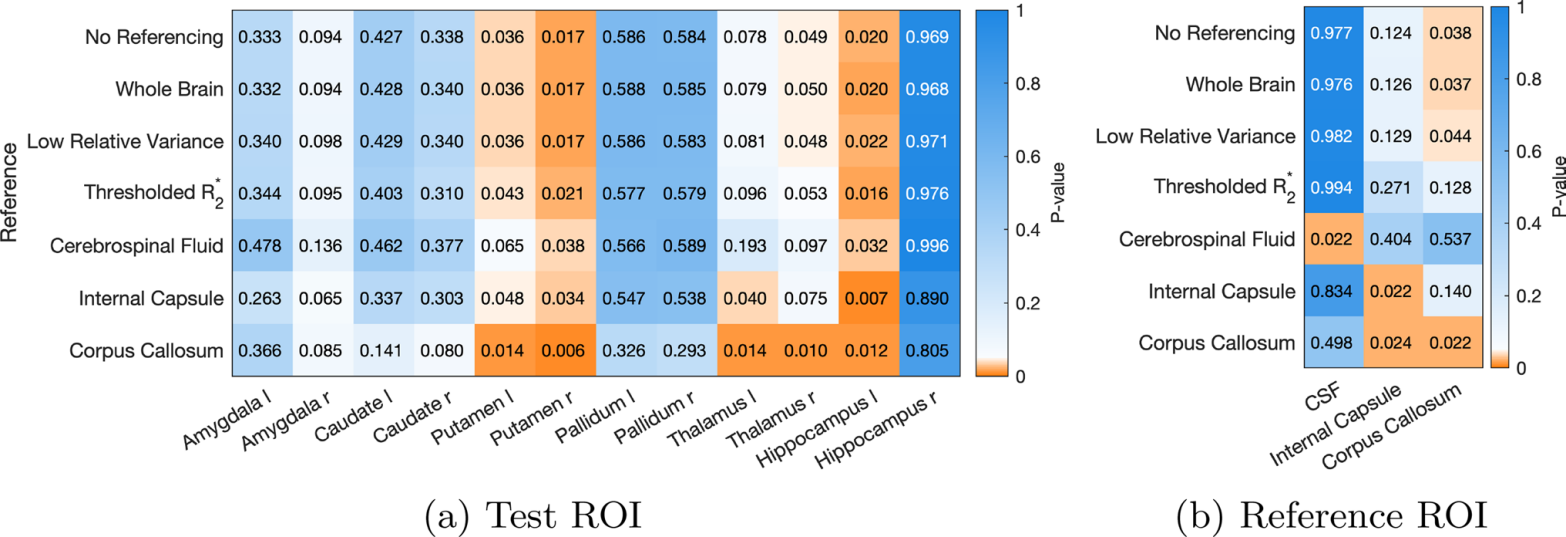
Groupwise ANOVA p-values in the epilepsy dataset using the different reference regions (y-axis) on anatomical ROI (a), and for the reference regions themselves (b). These indicate whether there is a significant between-group difference, but do not show which groups are different. P-values below 0.05 are indicated in orange and highlight where post-hoc between-group comparisons are expected to be statistically significant

**Fig. 6:**
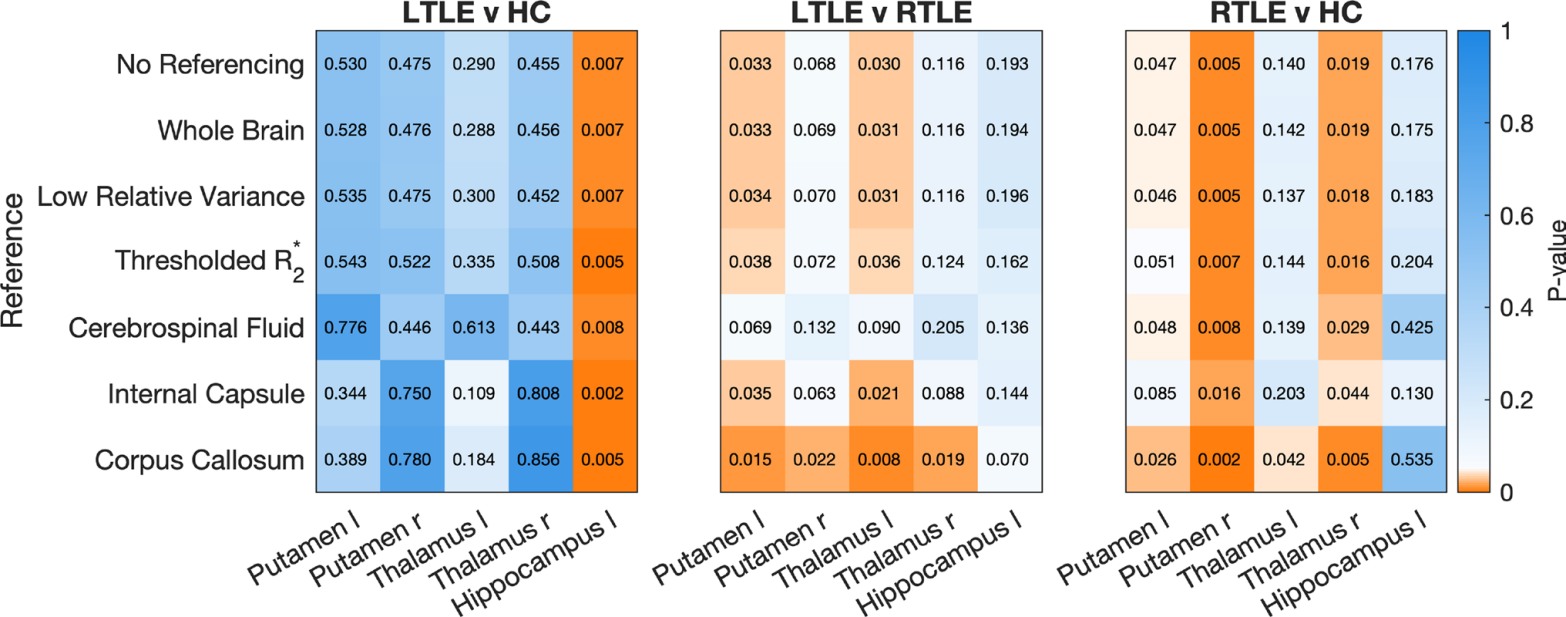
Tukey-Kramer multiple comparisons analysis of critical values for the different reference regions used in the epilepsy dataset. P-values below 0.05 are shown in orange highlight a significant difference in ROI mean susceptibility values between the two groups compared in each plot

Using the different reference regions for the epilepsy study, Figs. 5 and 6 show the groupwise (ANOVA) p-values, and the post-hoc t-test p-values for those regions of interest for which the ANOVA indicates significant between-group differences. In these figures all p-values<0.05 are coloured shades of orange to indicate significance. In Fig. 5b all reference regions show significant between-group differences when used as a reference, which makes sense given that their differences are essentially doubled. More important to note is that the corpus callosum has between group differences without a reference region being used, indicating potential pathological changes in the corpus callosum. In a clinical study, this would be a good reason to refrain from using that reference region, and it explains why referencing to the corpus callosum increases between-group differences (reducing the ANOVA p-value) for most test ROIs.

**Fig. 7:**
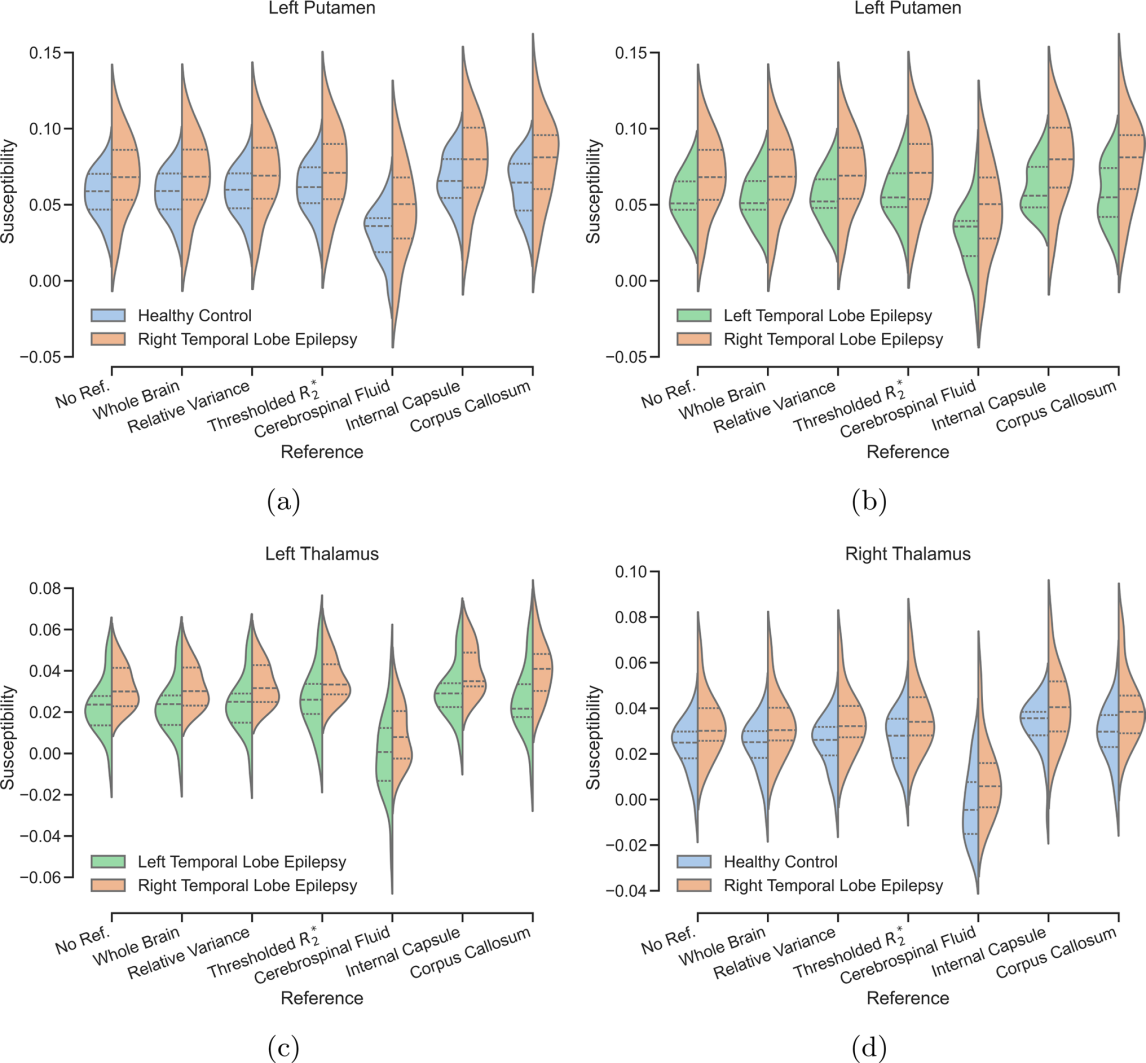
Violin plots of histograms of susceptibility distributions in subjects with right temporal lobe epilepsy (orange) compared to healthy controls (blue) and left temporal lobe epilepsy (green) using different reference regions in the left putamen, left thalamus and right thalamus in the epilepsy dataset. The dotted lines inside each violin plot denote the first and third quartiles of the distributions. The dashed line indicates the second quartile (mean)

Figure 7 provides an in-depth view of the changes in distributions for the ROIs with significant differences in the form of violin plots. We chose to highlight the left putamen and left and right amygdala because they show the largest variability between reference methods.

**Fig. 8:**
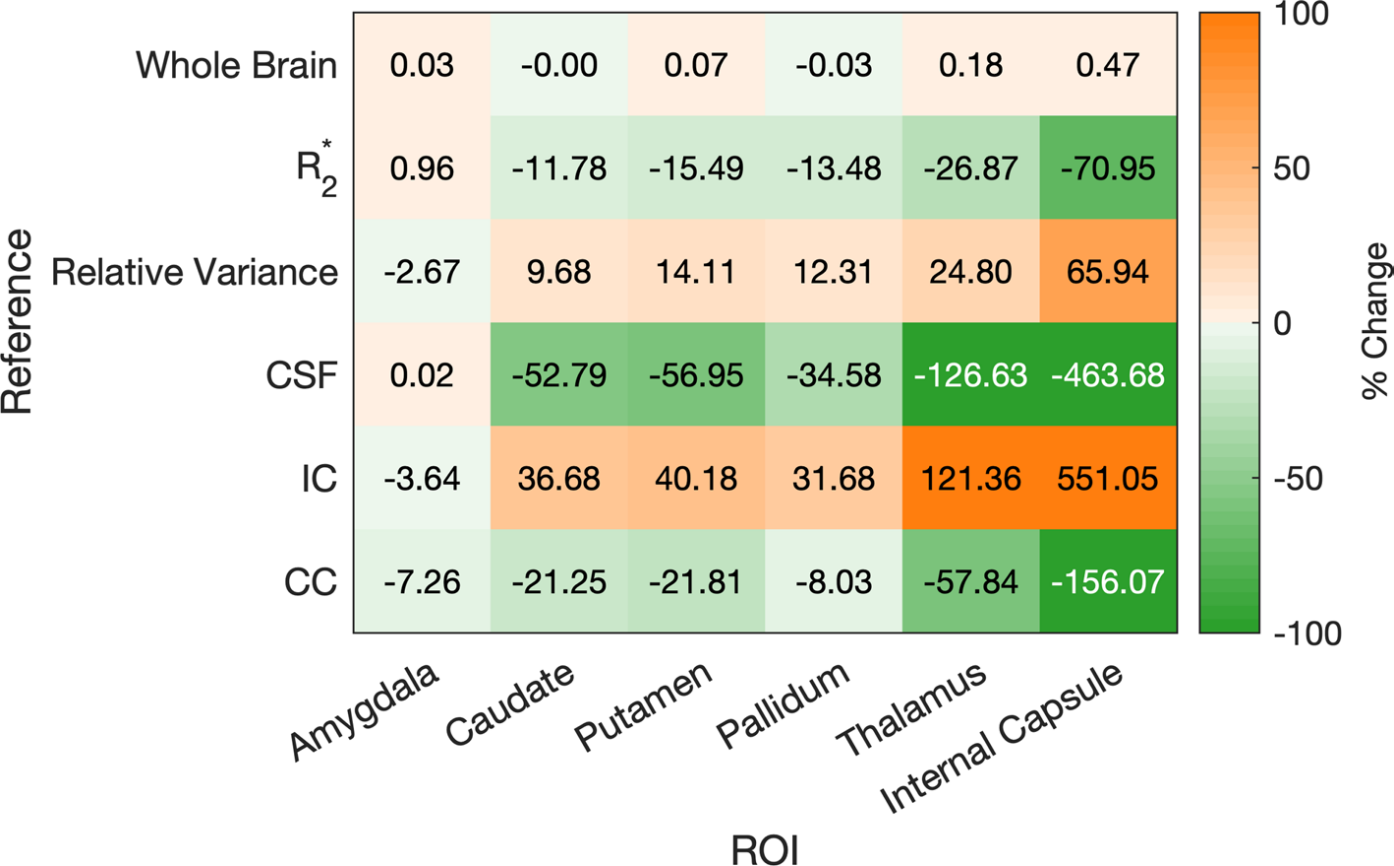
Epilepsy study percentage difference in ROI variance relative to no referencing: $\left( 100\times {\left[ {{\,\textrm{Var}\,}}(\textrm{ROI}-\textrm{ref} )-{{\,\textrm{Var}\,}}(\textrm{ROI} )\right] }/{{{\,\textrm{Var}\,}}(\textrm{ROI})}\right)$ depending on reference region used. Green denotes decreases in variance (increasing effect size) and orange denotes increases in variance (reducing the test strength)

Finally, to look at the overall influence of the reference regions on the test statistic we show the difference in variance for the test ROIs depending on reference region, compared to no explicit referencing, in Fig. 8.

## Discussion

From our theoretical investigation, we find that referencing will likely influence the variance of the across-subject test ROI mean (Eq. 8). Figure 8 verifies that this is indeed the case. Whole brain referencing has little impact here, which is to be expected as the whole brain mean values show the least variance across subjects: they are all very close to zero mean due to the background field removal and dipole inversion method used. CSF and $R_2^{*}$ -based regions both decrease the overall variance, improving the strength of the statistical test. This makes sense as both are based on voxels in similar brain regions. The white matter based relative variance ROI generally has a negative influence on the variance, which is interesting as it is a region of low variance (through the nature of its selection, as the region of least variance across the subjects), thereby indicating that it must have a low covariance (or negative covariance) with the rest of the ROI values to lead to this result. The corpus callosum improves the variance of all test ROIs, which would make it a great contender for a reference region were it not for the fact that, in this dataset, it is also correlated to the epilepsy status of the subject. Finally, the internal capsule negatively influences most test ROIs, due to its own large variance across subjects adding this “noise” to the overall dataset.

Before discussing the challenge dataset results, it is important to distinguish that in the synthetic dataset the distributions of ROI mean susceptibilities within non-independent reconstructions (“subjects”) were compared against the ground truth whilst in the epilepsy dataset of the distributions across subjects were compared between different groups.

The results of referencing the QSM 2.0 challenge dataset to different regions suggest that referencing can appear to improve the accuracy of some of the reconstructions (see Fig. 4 and Table 2). The number of accurately reconstructed ROIs (i.e. ROIs with no significant difference between their mean susceptibility and the ground truth value) is highest for the relative variance-based reference region at 47 accurate ROIs out of a maximum of 432 (all 9 regions of interest across all 48 submissions) possible (this is without adding the reference $^{\dagger }$ ROIs which are always accurate when referenced to). This clearly indicates the effect a reduction of variance can have on the test statistic. By decreasing the variance between the reconstructions through referencing a region that is correlated between the reconstructions, we improved the perceived accuracy. Whether this also reduces bias is more difficult to substantiate. That is, according to Equation (8), referencing to a region with relatively low variance and some covariance will reduce the overall variance and improve the accuracy in this specific simulated scenario.

The overall impact of using different reference regions on the epilepsy dataset is illustrated in Figs. 5 and 6. In Fig. 5 we can see that whole-brain and relative variance referencing do not change significant differences in any of the ROIs, whereas $R_2^{*}$-based referencing makes the group-wise differences in the right thalamus just insignificant (and the corpus callosum, but that is not one of the test ROIs). Larger differences are seen when using CSF and WM reference regions: CSF referencing reduces the number of significant ROIs overall, while referencing to the corpus callosum increases the number of significant ROIs (due to its correlation between groups, as noted in Fig. 5a), and referencing to the internal capsule makes the left thalamus significant instead of the right. That means that, for example, referencing to the CSF would lead to fewer ROIs with significant differences being found in the clinical study, and in a post-hoc t test.

In the epilepsy dataset, the ROIs found to show significant group-wise differences using ANOVA were analysed further using post-hoc Tukey–Kramer multiple-comparison tests. Referencing with different regions has less of an effect on the results of these post-hoc tests, with almost all referencing methods agreeing on most ROIs. Notable differences are CSF referencing, which reduced the difference between LTLE and RTLE patients, and referencing to the $R_2^{*}$ -based region or internal capsule reduced the significance of the RTLE and HC differences in the left putamen (Fig. 6). Referencing to the corpus callosum increases the number of ROIs with significantly different mean susceptibility values between the right and left temporal lobe epilepsy groups and RTLE and HC groups. In other words, referencing to this region could bias the results of this study by showing more ROIs with significant differences between these groups.

Finally, can this study suggest which reference region provides the most accurate results? Based on the epilepsy data, CSF seems like a robust reference region as it does not show any group-wise correlation (see Fig. 8) which would make it unlikely to introduce bias into the group-wise comparison. This reduction in significance due to CSF referencing gives us good confidence in the significant group-wise differences for the right putamen and left hippocampus (see Fig. 6), which are unaffected by reference region choice from a post-hoc point of view: all show significant differences at an uncertainty bound of 0.05. The same reasoning can be extended to the $R_2^{*}$ -based reference region as it is mostly a subset of the CSF albeit also incorporating CSF outside of the ventricles. Referencing to the $R_2^{*}$-based region would allow inclusion of the left putamen in the post-hoc analysis, for which the CSF-referenced and $R_2^{*}$-referenced results disagree, which would be an issue for clinical application of these results, as it suggests there may be bias involved in this “significant” result.

Based on the challenge dataset, the low relative variance based region would be another good choice as a reference. From a theoretical standpoint, the argument would be the opposite of that for CSF: with an increase in variance (as per Fig. 8) any improvement in statistical test result would stem from a reduction in bias, since the test statistic would be weaker in the first place. Interestingly, both the low relative variance region and the internal capsule led to more significant between group differences than CSF or $R_2^{*}$ -based referencing. This could then increase our confidence that those results from CSF or $R_2^{*}$ -based referencing results are indeed more accurate or less biased.

Finally, Fig. 7 highlights how CSF referencing, though seemingly the most “conservative” based on our analysis, can still have a big impact on the overall distribution of the data, and how a minor difference (i.e. in the left putamen in Fig. 7a where the distributions for the low relative variance and $R_2^{*}$- based reference regions for RTLE vs HC show minimal differences) can turn a significant finding into an insignificant one, as seen in Fig. 6 previously.

In all cases, when choosing reference regions, care must be taken not to fall into “p-hacking”, that is

“cherry-picking promising findings, also known by such terms as data dredging, significance chasing, significance questing, selective inference” [36]

Often, in clinical applications the more conservative approach may not lead to the most impressive publications: only showing two statistically significant ROIs instead of five sounds less impactful. However, this approach may lead to faster clinical translation, putting the majority of the effort into those regions with the largest effect size or realising more work needs to be done to get a precise or accurate enough measurement of biomarkers with a small effect size. Not explicitly referencing (or implicitly referencing to the whole brain volume, as these are essentially equivalent due to background field removal for all dipole inversion algorithms not including an explicit referencing term, see also the minute differences for the whole brain reference in Fig. 8) avoids having to choose an “optimal” reference region and prevents potential p-hacking. Not referencing can be seen as *safe* because it is unlikely to negatively affect the test statistic by increasing the number of false positives, although using a more specific region could provide more accurate results overall.

If a study is investigating particular pathology, referencing a carefully chosen region could be better. For example, if the clinical cohort has a very large age range, then choosing a reference region that is likely unaffected by age related changes (i.e. CSF [20]) would be better than reference regions that might exacerbate these effects. When dealing with a cohort that exhibits large anatomical changes (i.e. tumours or haemorrhages that affect a particular hemisphere or are widespread) using a non-anatomical reference region such as an $R_2^*$ -based one, for example, may be a robust choice.

It is important to choose reference region(s) in an a-priori fashion (before conducting a study) to prevent unintentional biasing of the results. It can be difficult to choose a reference region a-priori, especially when the effect of the pathology on magnetic susceptibility contrast may not be fully known. Therefore, implicit or full brain referencing minimizes confounding local correlations with pathology since one can reasonably assume that across the whole brain volume, the overall correlation with the pathology or effect of interest will be minimal compared to the inter-scan variability (bias) that referencing is designed to correct.

## Conclusions

This paper furthers our understanding of referencing in QSM, both from a theoretical, statistical point of view, indicating the expected differences in the mean and variance of the distribution being tested, and from a practical point of view, giving examples of how referencing can change the number of significant findings in a clinical study, or correct for dipole inversion method bias.

In the interest of preventing p-hacking or the introduction of bias we would recommend to always check the between-group correlation (using e.g. ANOVA) of the selected reference region(s), and to include the choice of reference region in the pre-registration of a clinical study. That is, when determining which regions of interest to test with which statistical test, to also include which reference region(s) are being considered, and why those regions are applicable for the pathology that is being investigated. In case no suitable reference region can readily be identified a-priori, not explicitly referencing (that is, implicitly referencing to the whole brain volume) can be considered a safe, conservative choice.

Finally, when publishing results, if the reference region values are shared together with the test ROI values it is easy for others to re-reference the results for each subject (i.e. add the reference means back and subtract a different reference mean) and perform additional statistical testing, this is also in line with the QSM consensus recommendations [4].

## Data Availability

The data that support the findings of this study are available upon reasonable request from the corresponding author.
